# Ego-Dokumente von Menschen mit Epilepsie im Nationalsozialismus

**DOI:** 10.1007/s00115-025-01865-z

**Published:** 2025-07-31

**Authors:** T. Baumann, A. Karenberg, H. Fangerau

**Affiliations:** 1 Institut für Geschichte und Ethik der Medizin, Universität zu Köln, Medizinische Fakultät und Universitätsklinikum Köln, Köln, Deutschland; 2 Institut für Geschichte, Theorie und Ethik der Medizin, Medizinische Fakultät, Centre for Health and Society, Heinrich-Heine-Universität Düsseldorf, Moorenstraße 5, Geb. 17.11 (Postfach 1114), 40225 Düsseldorf, Deutschland

**Keywords:** Epilepsie, 1933-1945, Patientenschicksale, Krankenakten, Ego-Dokumente, Epilepsy, 1933-1945, Patients’ fates, Medical records, Ego documents

## Abstract

**Hintergrund:**

Menschen mit Epilepsie gehörten im Nationalsozialismus zu einer stigmatisierten und verfolgten Gruppe. Trotzdem liegen nur wenige psychiatriehistorische Arbeiten vor, die sich gezielt mit der Lebenswirklichkeit dieser Gruppe im nationalsozialistischen Deutschland befasst haben.

**Ziel der Arbeit (Fragestellung):**

Im Sinne einer heuristischen Pionierstudie wird die Lebenswirklichkeit hospitalisierter Menschen mit Epilepsie in einer ausgewählten Einrichtung systematisch erforscht und dargestellt.

**Material und Methoden:**

Zahlreiche Patientenakten der „Heil- und Pflege-Anstalt Bonn“ wurden mittels der historisch-kritischen Methode untersucht. Etliche dieser Akten enthalten Briefe und andere Selbstzeugnisse der Patient(inn)en. Die vorliegende Studie konzentriert sich auf diese „Ego-Dokumente“ sowie auf Schreiben der Angehörigen.

**Ergebnisse:**

Während für die Patient(inn)en die Bewältigung des Alltags im Vordergrund stand, beschäftigten sich einige Verwandte eher mit der Frage nach der Vererbbarkeit der Epilepsie; andere, die ihren hospitalisierten Angehörigen helfen wollten, scheiterten nach 1939 zunehmend daran.

**Diskussion:**

Erst die bisher oft vernachlässigte Gesamtschau aller Quellentypen in Krankenakten (Anamnesebögen, Pflegeberichte, Ego-Dokumente und Briefe von Verwandten) ermöglicht ein annäherndes Verständnis der Situation hospitalisierter Menschen mit Epilepsie während der NS- und der Nachkriegszeit.

## Abstract (en)

**Background:**

People with epilepsy were a stigmatized and persecuted group under National Socialism. Nevertheless, there are only a few historical works that specifically address the reality of life for this group in Nazi Germany.

**Objective:**

In the sense of a heuristic pioneer study, the reality of life of in-patients with epilepsy in a selected institution is systematically researched and presented.

**Material and methods:**

Numerous patient files from the Bonn sanatorium (*Heil- und Pflege-Anstalt Bonn*) were analyzed using the historical-critical method. Some of these files contain letters and other testimonies from the patients. This study concentrates on these ego documents and on letters from relatives.

**Results:**

While coping with everyday life was central to the patients, some relatives were more concerned with the question of the hereditary nature of the epilepsy; others, who wanted to help their hospitalized relatives were increasingly unable to do so after 1939.

**Conclusion:**

Only an overall view of all types of sources in medical records (medical history forms, nursing reports, ego documents and letters from relatives), which has so far often been neglected, enables an approximate understanding of the situation of hospitalized patients with epilepsy during the National Socialist era and the post-war period.

## Hintergrund

**Abb. 1:**
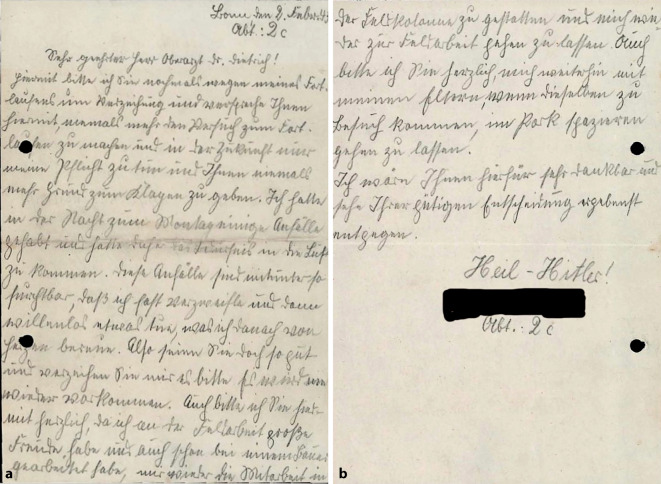
Eingabe eines 17-Jährigen im Jahr 1943 von der Hand eines Mitpatienten. **a** Seite 1, **b** Seite 2 (Psychiatriemuseum Ver-rückte Zeiten Bonn, Bestand 04‑F, individualisierte Treffernummer 16, S. 53f.)

Wie kaum eine andere historische Quellengattung spiegeln Ego-Dokumente den Alltag und das individuelle Erleben betroffener Personen. Ein Beispiel bietet das Schreiben eines 17-jährigen Patienten vom 2. Februar 1943 an einen Oberarzt der Heil- und Pflegeanstalt Bonn:

„Hiermit bitte ich Sie nochmals wegen meines Fortlaufens um Verzeihung _[_…_]_ Ich hatte in der Nacht zum Montag einige Anfälle gehabt und hatte daher das Bedürfnis_[,]_ in die Luft zu kommen. Diese Anfälle sind mitunter so furchtbar, daß ich fast verzweifle und dann willenlos etwas tue, was ich danach von Herzen bereue“ (Abb. 1, 04-F-16: 53f.).

Menschen mit Epilepsie gehörten im Nationalsozialismus (NS) zu einer besonders stigmatisierten Gruppe, waren sie doch mit Inkrafttreten des „Gesetzes zur Verhütung erbkranken Nachwuchses“ von einer Zwangssterilisation und nach Beginn der sogenannten *Euthanasie*-Aktionen vom Tod bedroht [1, 8 S. 498f.]. Bisher kamen speziell diese Menschen bei der Darstellung ihres Schicksals in der NS-Zeit allerdings kaum [7] oder jedenfalls nicht systematisch zu Wort [9 S. 123–132, 226–240, 20, 24]. Der Fokus der Forschung lag auf zeitgenössischen neuropsychiatrischen Fachdiskursen [13, 14] oder ausgewählten „Täter“-Biografien [3, 11]. In Arbeiten zur Zwangssterilisation [4, 10, 12, 17, 18, 22] bildete Epilepsie keinen Schwerpunkt, Studien zu Heilanstalten konzentrierten sich auf statistische Erhebungen [6, 15 S. 71–118, 21]. Somit steht eine „Annäherung an den Menschen“ [19], der von einer in der damaligen Zeit kaum behandelbaren Epilepsie betroffen war, für die Zeit von 1933 bis 1945 noch weitgehend aus.

Um sich dieser Lücke anzunähern, werden hier „Ego-Dokumente“ untersucht – insbesondere, um zu verhindern, dass möglichen Tätern über die von ihnen verfassten Dokumente auch noch das letzte Wort bleibt. Hier sollen nun typische Parameter des Lebens von Menschen mit Epilepsie in der NS-Zeit skizziert werden. Dazu werden Quellen herangezogen, die aussagekräftig die Interaktion von Patient(inn)en mit Behandelnden, Mitpatient(inn)en und Angehörigen spiegeln.

## Methodik

Als Quellenbasis der vorliegenden Studie dienen Krankenakten der ehemaligen Provinzial-Heil- und Pflegeanstalt (PHPA) Bonn, Vorgängerin der heutigen LVR-Klinik Bonn. Die in den 1870er-Jahren entstandene Einrichtung war nach 1933 in die NS-Eugenik und NS-*Euthanasie* einbezogen. Ihr Direktor Kurt Pohlisch machte Bonn bald zu einem Zentrum der eugenischen Forschung mit Epilepsie als einem besonderen Schwerpunkt [8 S. 481–492, 16, S. 1f.].

Akten konnten von November 2022 bis Februar 2024 im dortigen Psychiatrie-Museum eingesehen werden. In den Beständen 04 (Männer 1933–1955), 05 (Frauen 1933–1955) und 06 (Männer und Frauen 1956–1971) wurden repräsentativ die Akten aller Patient(inn)en gesichtet, deren Nachnamen mit B, F oder H beginnen. Diese 9 Stichproben umfassen insgesamt 5019 Personen. Einige davon waren schon zu einer Zeit vor dem im Bestandsnamen jeweils genannten Zeitraum erstmals in der Anstalt.

Da Diagnosen sich manchmal änderten, mussten alle Akten komplett durchgesehen werden. Detailliert ausgewertet wurden nur Akten mit Begrifflichkeiten wie Epilepsie, „Fallsucht“ oder „Krampfanfälle“. Inkludiert sind dabei neben Patient(inn)en mit einer entsprechenden Diagnose auch Menschen, die von einem Erbgesundheitsgericht (EGG) eingewiesen worden waren, um klären zu lassen, ob eine vererbliche Epilepsie im Sinn des „Gesetzes zur Verhütung erbkranken Nachwuchses“ vorlag. Dem gesamten Epilepsiebereich (samt zeitweiliger Zuschreibung einer solchen Erkrankung) sind insgesamt 267 Patientinnen und Patienten (5,3 %) zuzuordnen. Dieser Anteil ist kleiner als die in den „Reichsirrenstatistiken“ von 1934 und 1935 für Anstalten (ohne Krankenhäuser) genannten 9,9 und 9,6 % [26 S. 230]. Tatsächlich besaß der Standort Bonn nur in den Jahren um 1940 eine besondere Expertise für die Diagnose vererblicher Epilepsie [2].

Nur ein Teil der Akten befasst sich mit Epilepsie. Beispielsweise sind dies in der Stichprobe 04‑B (Männer, deren Nachnamen mit B beginnen) die Krankenakten von 77 Personen (6,7 %) unter 1157 Patienten. Zur Anonymisierung sind sie hier mit 04-B‑1 bis 04-B-77 benannt. 18 davon waren in der NS-Zeit hospitalisiert *und* hinterließen Ego-Dokumente wie Briefe oder Tagebucheinträge. Die Verhältnisse in den anderen Stichproben liegen ähnlich. Über Briefe und Tagebücher hinaus wurden *alle* Selbstzeugnisse berücksichtigt, also „auch unfreiwillige Aussagen aus Verhörprotokollen und Akten“ [5, S. 361].

## Ergebnisse

### Aufnahme und Vorgeschichte

Der Aufenthalt in der Bonner Anstalt war für die Patient(inn)en oft nicht freiwillig. Den klinischen Blickwinkel des Aufnahmevorgangs spiegeln Formulare wider, die Ärzte ausfüllten und dabei Angaben von Patient(inn)en und ggf. begleitenden Verwandten oft genug zweifelnd wiedergaben. So wurde eine immer kritisch zu hinterfragende Aktenfigur konstruiert. Die 1906 geborene Frau B. etwa galt schon bei ihrer Erstaufnahme in Bonn als „erbkrank“ und war bereits 4 Jahre zuvor sterilisiert worden. Sie wurde im März 1939 von der Universitäts-Nervenklinik Köln-Lindenburg überwiesen „wegen epileptischer Anfälle mit Wesensänderung und Erregungszuständen“ (05-B-44: 3f./63–66). Bonner Ärzte protokollierten kurz darauf eine „*epileptische Wesensänderung*“:

„Die Kranke ist klebrig, [s]ie hängt sich mit allerlei Fragen dauernd an den Arzt und an die Pflegerinnen und man weiss sie oft geradezu nicht loszuwerden. Dabei merkt sie selbst garnicht, dass sie lästig fällt durch ihre andauernd gestellten Fragen _[…]_ wie sie über ihre intimen Erlebnisse berichtet, zeugt von einem nicht unbedeutenden Verlust feinerer ethischer Empfindungen, _[…]_“ (05-B-44: 10–12).

Da bei der Erstaufnahme einer Person kein Vergleich zu deren prämorbider Persönlichkeit zu ziehen war, führten Einträge wie dieser zu einer Verknüpfung von Aussagen zu Symptomatik und Diagnose mit oft negativen Zuschreibungen (siehe auch [16, S. 36–40]). Diese wertende Betrachtungsweise hielt sich lange – manchmal noch Jahrzehnte (z. B. 06-H-2: 69/166).

### Vor und nach der „Unfruchtbarmachung“

In den ausgewerteten Akten fanden sich wenig Proteste gegen eine drohende Sterilisation wegen Epilepsie. Der Psychiater Werner Villinger (Leitender Arzt der Anstalt Bethel), selbst tief verstrickt in die NS-Politik der Zwangssterilisation, behauptete 1935, dass „die Mehrzahl der Epileptischen“ sich „durch die Operation zu Menschen zweiter Klasse“ herabgesetzt fühlten und mit großer „Zähigkeit“ widersprachen, sich aber zumeist dem nötigen „Opfer“ beugten [23, S. 83]. Ein Bonner Beispiel bietet das Schreiben eines Stiefvaters eines 21-Jährigen aus dem Jahr 1934. Er hielt fest, dass sein Sohn nicht erbkrank, sondern unter harter Arbeit zusammengebrochen sei; doch hatte diese Intervention keinen Erfolg (04-B-10: 43/47f.). Ein Anfang Juli 1940 aus der PHPA entlassener Mann, dessen Sterilisation das EGG Bonn „wegen angeb[orenem] Schwachsinn und erbl[icher] Fallsucht“ später im Monat „beschloss“, beschwerte sich im September, allerdings weil er laut Gesundheitsamt die Kosten tragen müsse. Ihm wurde vom Arzt versichert, dass das Wohlfahrtsamt zahle (04-H-12: 3/16/26/66f.).

Eine Entlassung im Vorfeld der „Unfruchtbarmachung“ – mit Zustimmung des zuständigen Gesundheitsamtes – hatte allerdings Seltenheitswert und deutet auf Bevorzugung eines aus Sicht der Ärzte offenkundig ungewöhnlich einsichtigen Patienten hin (04-H-12: 25). Sonst kamen Frauen meist direkt in die Bonner Universitäts-Frauenklinik (z. B. 05-B-37: 144, 05-B-47: 10), Männer in die Chirurgische Klinik (z. B. 04-H-14: 27) und danach oft wieder in die PHPA Bonn zurück. In einem anderen Fall bat die PHPA im Juli 1941 erfolglos um die Entlassung vor der vom EGG Bonn bereits angeordneten „Unfruchtbarmachung“ (04-B-34: 12). Die ausgewerteten Ego-Dokumente beschreiben die Operation nicht. Doch eine Frau mit Epilepsie, die 1936 in der I. Universitätsfrauenklinik München sterilisiert worden war, schilderte Jahrzehnte später, dass sie und ihre Eltern aus Angst vor vermuteter Strafe („Dachau“) dem Eingriff zugestimmt hatten. In beiden Leisten habe sie danach Schnitte gehabt. Und noch in der Klinik musste sie eine Schweigeverpflichtung unterzeichnen [10, S. 101–103].

Nicht nur hatten Patient(inn)en nachweislich Sorge vor den Folgen einer Operation (05-B-18: 25–28). Jede Äußerung im Klinikumfeld konnte sich zudem auf Familienangehörige auswirken – die im Falle der Annahme einer hereditären Erkrankung ebenfalls mitbetroffen waren. 1937 informierte der damalige Assistenzarzt Josef Gierlich umgehend das EGG, als der Bruder eines Patienten von weiteren Epilepsiefällen in der Familie berichtet hatte (04-H-7: 22). Auch „Gesunde“ aus „Epileptikersippen“ waren mitbetroffen [3, S. 32f.]: Der Schwager einer 1902 geborenen Frau fragte 1938 sogar besorgt bei der Bonner Heilanstalt an, ob es sich um ein erbliches Leiden handle und er folglich mit der Schwester der Patientin keine Kinder haben solle (06-H-1: 169f.). Der Bruder, ein Arzt, war tatsächlich schon 1937 informiert worden, dass weder Liquor noch Pneumenzephalogramm auf eine „traumatische Entstehung der Epilepsie“ hindeuteten (06-H-1: 21). Ein Erbgesundheitsverfahren wird in der Akte überraschenderweise dennoch nicht erwähnt; vielmehr bescheinigte die Anstalt dieser Frau im April 1942, dass sie „nicht an erblicher Fallsucht leidet“ (06-H-1: 152). Die Pflegeberichte ordneten ihr 1938 bis 1945 Fleiß und Ruhe zu (06-H-1: 137f.). Patient(inn)en, die sich nicht so geordnet verhielten, sondern Erregungszustände zeigten, wurden dagegen ruhiggestellt und trugen möglicherweise ein höheres Risiko, sterilisiert zu werden.

### Medizinische Behandlung

Als Antiepileptikum wurde meist Luminal genutzt, das oft sedierend wirkte [14, S. 101f.]. Pfleger setzten falls notwendig die Einnahme mit „Zureden“ durch (04-B-60: 141). Als die bereits genannte Frau B. im November 1941 wieder in die PHPA Bonn eingewiesen wurde (05-B-44: 38), schilderte ihre Mutter dem dortigen Oberarzt Werner einen Suizidversuch ihrer Tochter und bat:

„Da meine Tochter nach meiner Ansicht noch mehr durch das jahrelange Einnehmen von Luminal, als durch ihre Krankheit so sehr verelendet ist, möchte ich sehr bitten, ihr doch kein Luminal mehr zu verordnen. Besonders aus dem Grunde_[,]_ weil Luminal so lange-genommen ihre Anfälle gar nicht herabsetzt, und ihr anderseits sehr schadet“ (05-B-44: 122f.).

Die Situation verschlechterte sich aber weiter: Kurz darauf wandte sich die Mutter an einen anderen Arzt und mutmaßte, dass die schlechte Verfassung ihrer Tochter unter anderem daher rühre, dass sie „im Bett festgebunden wird“. Die Pflegerinnen gäben ihr auch nicht genug zu Trinken; der Arzt solle anordnen, ihr „öfter mal einen Schluck Wasser“ zu geben (05-B-44: 126f.). Drei Tage später starb Frau B. – nur 10 Tage nach ihrer Wiederaufnahme. Die Sektion erfolgte unmittelbar nach dem Tod. Der Sektionsbericht nennt „Lungenembolie und Bronchopneumonie“ als Todesursache und hält fest: „Dürftiger Allgemeinzustand“ (05-B-44: 3/57).

**Abb. 2:**
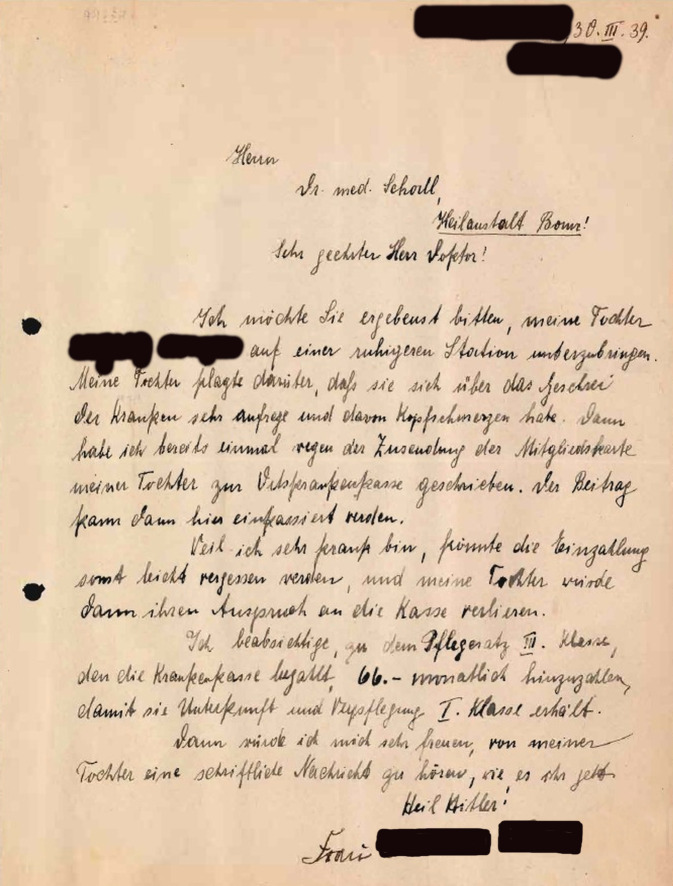
Schreiben der Mutter von Frau B. im März 1939 an einen Arzt der PHPA (Psychiatrie-Museum Ver-rückte Zeiten Bonn, Bestand 05‑B, individualisierte Treffernummer 44, S. 30)

Bei ihrem ersten Aufenthalt war Frau B. noch ganz anders behandelt worden; vor dem Krieg spielte ein gehobener sozialer Status noch eine Rolle. Ihre Mutter hatte im März 1939 um die Verlegung auf eine „ruhigere Station“ gebeten (Abb. 2); dem war „versuchsweise“ entsprochen worden, trotz anfänglicher Unruhe der Patientin (05-B-44: 30f./75). Auf Fragen eines Arztes zur „Vorgeschichte“ gab Frau B. an, 1932 ein Examen als Diplomvolkswirtin gemacht zu haben (05-B-44: 10). Einer ihrer Brüder, ein promovierter Jurist, beteiligte sich, wie er im April 1939 schrieb, an den Unterbringungskosten und bemühte sich mit weiteren Geschwistern und der Mutter um die Entlassung (05-B-44: 34f./78f.). Nachdem dies Erfolg hatte, bedankte sich Frau B. im Juni überschwänglich bei Oberarzt Werner dafür, was er ihr „alles Gute getan“ habe (05-B-44: 99f.).

Auffällig bei Frau B. ist, dass ihre Angehörigen 1941 keine anderweitige Unterbringung mehr organisieren wollten oder konnten. Bei anderen Patient(inn)en sind dagegen ab 1939 von Angehörigen unterschriebene „Revers“ zur Entlassung auf eigene Verantwortung erhalten (z. B. 04-B-8: 27 [11.01.1944]). Die Motivation wird aus den Krankenakten kaum ersichtlich; doch könnte die Absicht bestanden haben, Patient(inn)en in Sicherheit zu bringen. Im Vorfeld der im Krieg durchgeführten Transporte von Kranken in Tötungsanstalten informierten Bonner Mediziner manche Verwandte laut Forschungsstand sogar vor einer drohenden Verlegung – und andere möglicherweise nicht [1 S. 71, 8 S. 498f., 12, S. 299].

Auch zur Frage einer „dezentralen ‚Euthanasie‘“ [25] werfen die eingesehenen Bonner Krankenakten allenfalls Verdachtsfälle auf. Im Dezember 1941 jedenfalls bestätigte die Staatsanwaltschaft Köln den Tod der eingangs genannten „an Seelenstörung leidenden“ Frau B. einfach per ausgefülltem Vordruck (05-B-44: 129). Möglicherweise parallel zu sehen ist eine Patientin, deren „Unfruchtbarmachung“ das EGG Bonn 1934 beschlossen hatte (05-B-11: 30f.) und die im Dezember 1944 in der PHPA neuerlich aufgenommen wurde. Im ersten Pflegeberichteintrag steht: „Dranghaft unruhig“ und „Luminal“, 2 Tage später „Sondenfütterung“, am Folgetag „bis 37,9°“ und 3 Tage später „Exitus“ (05-B-11: 41/61). Ob eine gezielte Tötung etwa durch Barbituratüberdosierung herbeigeführt wurde [2, 25], bleibt wiederum unklar.

### Beschäftigung und Konflikte

Wie die vorgestellten Zeugnisse zeigen, geben Ego-Dokumente von Patient(inn)en einen zwar schlaglichtartigen, dennoch unverzichtbaren und bislang vernachlässigten Einblick in den Anstaltsalltag. Eine Patientin schrieb:

„Die Schwestern sind manchmal nicht so grausam, sie werden durch Anordnungen der Ärzte veranlaßt_[,]_ grausam zu sein“ (05-B-18: 25–28).

Ein Schreiben des eingangs zitierten 17-Jährigen beleuchtet eindrucksvoll sowohl den auf Erkrankte ausgeübten Druck, Arbeitsleistungen zu erbringen, als auch Konflikte und Gewalt zwischen Patienten. Am 9. Februar 1943 berichtete er einem Oberarzt über einen Vorfall bei der Feldarbeit und bat um Schutz – die Zeilen hatte wieder ein Mitpatient niedergeschrieben:

„Beim Aussuchen von faulen Kartoffeln übersah ich einen Solchen und da schlug mich der Patient _[…]_, Abt. 2e/Dr. Gierlich, auf den Kopf und schlug mich mit der Faust ins Gesicht und schlug mir die Wunden am Hinterkopf auf, sodaß ich Kopfschmerzen bekam. Ich möchte noch dabei bemerken, daß ich vergangene Nacht einen Anfall gehabt hatte!“ (04-F-16: 55f.).

Im Herbst 1943 verfasste der mittlerweile 18-jährige Patient – nun selbst – ein weiteres Schreiben, weil er neuerlich geflohen war und nicht mehr „mit in die Kolonne gehen“ durfte. Bei einem Besuch habe seine Mutter gesagt, „wenn ich noch ein mal die Anstalt entlaufen werde, wäre für mi[ch] das Eltern Haus endgültig geschlossen“ (04-F-16: 11). Die Gründe seiner wiederholten Flucht lassen sich über die Akte nicht näher rekonstruieren. Die knappen Pflegeberichte enden Anfang 1946 mit dem Eintrag: „Exitus infolge Herzschwäche bei Status epilepticus“ (04-F-16: 82).

Reaktionen von Angehörigen auf den Tod von Patient(inn)en sind in den ausgewerteten Akten sehr wenige erhalten. Bei einer im April 1943 in Bonn mit „Erbliche[r] Fallsucht“ wiederaufgenommenen Kranken, die schon nach 7 Tagen verstarb (05-H-3: 21), suchte der von ihrem „unerwartet schellen Tod“ schockierte Vater die ihm offenbar angekündigte Feuerbestattung zu verhindern (05-H-3: 16). Eine Antwort ist nicht überliefert.

## Diskussion

In vielen medizinhistorischen Arbeiten zur NS-Zeit werden Patient(inn)en nur als Zahlen abgebildet, was die historische Erforschung medizinischer Verbrechen im NS von den betroffenen Menschen entfernt.

Die Relevanz von Ego-Dokumenten ergibt sich daraus, dass sie es ermöglichen, die Situation von Patient(inn)en besser zu erfassen. Auch wenn sie vieles nur schlaglichtartig beleuchten, enthalten sie doch (1) Informationen, die in allen anderen Dokumenten fehlen; und sie bieten (2) eine Perspektive, die Leben und Leiden von Patient(inn)en umfassender erschließt. Die Aussagekraft der vorgefundenen Ego-Dokumente ist uneinheitlich. Manche Patient(inn)en fragten in Briefen nur nach verlorenen Unterlagen. Einen Teil der Selbstzeugnisse fertigten Betroffene auf Anordnung der Ärzte an, insbesondere Lebensläufe für Intelligenztests mit den ihnen eigenen Biografiekonstruktionen.

Bei der Auswertung von Ego-Dokumenten ergeben sich auch einige Einschränkungen: Einige Patient(inn)en – besonders solche mit sehr vielen Anfällen – hinterließen kaum Selbstzeugnisse; sie bleiben unterrepräsentiert. Schreiben von Verwandten, die in etlichen Akten erhalten sind, können dies immerhin teilweise ausgleichen. Bei manchen handelt es sich um sehr aussagekräftige Schreiben. Insgesamt ermöglicht demnach nur eine Gesamtschau aller Quellentypen in den Krankenakten (Anamnesebögen, Pflegeberichte, Ego-Dokumente und Briefe Angehöriger) eine Annäherung an den Menschen in der Geschichte.

## Fazit für die Praxis

- Ergänzend zu Krankenakten, die aus der institutionellen Perspektive geschrieben wurden, beleuchten Ego-Dokumente und Angehörigenschreiben den Alltag von Menschen mit Epilepsie aus deren Sicht im Sinne historischer Momentaufnahmen.
- Patient(inn)en treten vermittelt durch Ego-Dokumente als aktive historische Subjekte in Erscheinung, wenn sie etwa in Konfliktfällen das Wort ergriffen.
- Je weniger sich Patient(inn)en oder ihre Angehörigen äußern konnten – etwa infolge der/einer Krankheit – desto mehr waren sie ärztlicher und pflegerischer Willkür ausgeliefert.

## Data Availability

Alle Daten, die die Ergebnisse dieser wissenschaftlichen Publikation stützen, sind innerhalb des vorliegenden Artikels mit Quellenangaben belegt.

## Abkürzungen

Diss.: (ungedruckte) Dissertation
EGG: Erbgesundheitsgericht (in Bonn etc.)
f.: folgende Seite
LVR: Landschaftsverband Rheinland (seit 1953)
NS, NS-: Nationalsozialismus, nationalsozialistisch _[…]_
PHPA: Provinzial-Heil- und Pflegeanstalt (in Bonn etc.)

## References

[1] Aly G (2013) Die Belasteten. ‚Euthanasie‘ 1939–1945. Eine Gesellschaftsgeschichte. 2. Aufl. S. Fischer, Frankfurt a. M.

[2] Baumann T, Karenberg A, Fangerau H (im Druck) Bonner Ärzte und Menschen mit Epilepsie im Nationalsozialismus. Nervenheilkunde (Suppl) 10.1007/s00115-025-01865-z PMC13171986 40742442

[3] Baumann T, Sparing F, Martin M et al (2020) Neurophysiologen im Nationalsozialismus – Hans Berger, Paul Hoffmann, Richard Jung und Alois E. Kornmüller. Klin Neurophysiol 51:14–41

[4] Bock G (2010) Zwangssterilisation im Nationalsozialismus. Studien zur Rassenpolitik und Geschlechterpolitik. 2. Aufl. Monsenstein und Vannerdat, Münster

[5] Brückner B, Röske T, Rotzoll M et al (2019) Geschichte der Psychiatrie „von unten“. Entwicklung und Stand der deutschsprachigen Forschung. Med Hist J 54:347–376

[6] Cranach M, Siemen HL (2012) Psychiatrie im Nationalsozialismus. Die Bayerischen Heil- und Pflegeanstalten zwischen 1933 und 1945. 2. Aufl. Oldenbourg, München

[7] Englisch S (2001) Die Anwendung des „Gesetzes zur Verhütung erbkranken Nachwuchses“ auf Epilepsiekranke in Bethel 1933–1945. Diss, Münster

[8] Forsbach R (2006) Die Medizinische Fakultät der Universität Bonn im „Dritten Reich“. R. Oldenbourg, München

[9] Fuchs P, Rotzoll M, Müller U et al (2007) „Das Vergessen der Vernichtung ist Teil der Vernichtung selbst“. Lebensgeschichten von Opfern der nationalsozialistischen „Euthanasie“. Wallstein, Göttingen

[10] Horban CT (1999) Gynäkologie und Nationalsozialismus. Die zwangssterilisierten, ehemaligen Patientinnen der I. Universitätsklinik heute – eine späte Entschuldigung. Diss., Ludwigs-Maximilians-Universität München

[11] Hulverscheidt M, Kaminsky U (2022) Der Chirurg der von Bodelschwinghschen Anstalten Bethel. Richard Wilmanns (1880–1958) – medizinhistorische Erkenntnisse und deren veränderte Wahrnehmung. In: Rauh P, Voggenreiter M, Ude-Koeller S et al (Hrsg) Medizintäter. Ärzte und Ärztinnen im Spiegel der NS-Täterforschung. Böhlau, Köln, S 203–227

[12] Klein AS (2020) „Euthanasie“, Zwangssterilisationen, Humanexperimente. NS-Medizinverbrechen an Rhein und Sieg 1933–1945. Böhlau, Wien

[13] Martin M, Fangerau H, Karenberg A (2016) Neurologie und Neurologen in der NS-Zeit. Das Beispiel der Epilepsieforschung. Nervenarzt 87(Suppl 1):18–29 27325159 10.1007/s00115-016-0141-x

[14] Möller T (2010) Vom wissenschaftlichen Wissen zum gesellschaftlichen Vorurteil. Erblichkeit und Psychopathologie im deutschen Epilepsiediskurs. Mabuse, Frankfurt a. M.

[15] Müller T (2005) Untersuchungen zum Schicksal von Patienten mit Epilepsie in der Zeit des Nationalsozialismus von 1933–1945 am Beispiel ihrer Betreuung und Behandlung in der Landesheilanstalt Altscherbitz. Diss, Leipzig

[16] Pohlisch K (1940) Die erbliche Fallsucht. Allgemeiner und klinischer Teil. In: Gütt A, Rüdin E (Hrsg) Handbuch der Erbkrankheiten, Bd. 3. Thieme, Leipzig, S 1–102

[17] Ruckert F (2012) Zwangssterilisationen im Dritten Reich 1933–1945. Das Schicksal der Opfer am Beispiel der Frauenklinik des Städtischen Krankenhauses und der Hebammenlehranstalt Mainz. Franz Steiner, Stuttgart

[18] Schmacke N, Güse HG (1984) Zwangssterilisiert. Verleugnet – vergessen. Zur Geschichte der nationalsozialistischen Rassenhygiene am Beispiel Bremen. Brockkamp, Bremen

[19] Schulze W (1996) Ego-Dokumente. Annäherung an den Menschen in der Geschichte? In: Schulze W (Hrsg) Ego-Dokumente. Akademie Verlag, Berlin, S 11–30

[20] Söhner F, Cranach M, Fangerau H et al (2017) Nach der „Aktion T4“. „Regionalisierte Euthanasie“ in der Heil- und Pflegeanstalt Günzburg. Nervenarzt 88: 1065–1073 27531209 10.1007/s00115-016-0190-1

[21] Sonntag EM (2020) Die Zwangssterilisationen in der Hessischen Hebammenlehranstalt Mainz im Dritten Reich. Die klinische Umsetzung und die Verantwortung des leitenden Personals. Diss, Mainz

[22] Venza-Tillmanns J (2017) „… ist unfruchtbar zu machen“. Zum Thema Zwangssterilisation aus einigen Krankenakten der Provinzial Heil- und Pflegeanstalt Bonn und der Dr. Herz’schen Privatklinik. Psychiatrie-Museum Ver-rückte Zeiten, Bonn

[23] Villinger W (1935) Erfahrungen mit dem Erbkrankheitenverhütungsgesetz. Zeitschr F Psych Hyg 8:70–85

[24] Völker O (2022) Zwangssterilisation an Psychiatrieinsassinnen in Mainz 1933–1945. Die Lebensgeschichten der betroffenen Frauen der Heil- und Pflegeanstalten Alzey und „Philippshospital“ Riedstadt Goddelau. V&R unipress, Göttingen

[25] Voggenreiter M, Ude-Koeller S, Rettig D et al (2024) Verhungert? Vernachlässigt? Die Opfer der dezentralen „Euthanasie“ in der Heil- und Pflegeanstalt Erlangen. Freund S, Rettig D, Ude-Koeller S et al (Hrsg) NS-„Euthanasie“ in Franken. Die Heil- und Pflegeanstalt Erlangen, die „Aktion T4“ und das „Hungersterben“. Bd. 1. Böhlau, Köln, S 169–226

[26] Wunder M (1992) Euthanasie in den letzten Kriegsjahren. Die Jahre 1944 und 1945 in der Heil- und Pflegeanstalt Hamburg-Langenhorn. Matthiesen, Husum

